# Spectral CT imaging in colorectal cancer: current applications, limitations, and future perspectives

**DOI:** 10.1186/s13244-026-02212-9

**Published:** 2026-02-09

**Authors:** Rémi Grange, Mathilde Wagner, Nazim Benzerdjeb, Olivier Glehen, Vahan Kepenekian, Salim Si-Mohamed, Pascal Rousset

**Affiliations:** 1 Department of Radiology, University Hospital of Saint-Etienne, Saint-Priest-en-Jarez, France; 2 CICLY, EMR 3738, Lyon 1 University, Lyon, France; 3 Department of Radiology, Hopital Saint-Antoine, Paris, France; 4 Department of Pathology, Centre Hospitalier Lyon-Sud, Hospices Civils de Lyon, Pierre-Bénite, France; 5 Department of General Surgery & Surgical Oncology, Centre Hospitalier Lyon-Sud, Hospices Civils de Lyon, Pierre-Bénite, France; 6 University of Lyon, INSA-Lyon, Université Claude Bernard Lyon 1, UJM-Saint Etienne, CNRS, Inserm, CREATIS UMR 5220, U1206, F-69621, Villeurbanne, France; 7 Department of Radiology, Louis Pradel Hospital, Hospices Civils de Lyon, Bron, France; 8 Department of Radiology, CHU Lyon-Sud, Hospices Civils de Lyon, Pierre-Bénite, France

**Keywords:** Spectral CT, Colorectal cancer, Liver metastases, Peritoneal metastases

## Abstract

**Abstract:**

Colorectal cancer (CRC) is the third most common malignancy worldwide, and early detection is vital to prevent metastasis and postoperative recurrence. This review summarizes current applications of spectral computed tomography (CT) in CRC, including its principles, spectral parameters used for evaluating primary and metastatic lesions, and key findings from recent literature. A systematic search of PubMed, Web of Science, and Google Scholar identified English-language studies published between April 2018 and April 2025 using the keywords: “spectral CT,” “spectral imaging,” “dual-layer spectral CT,” “dual-energy spectral CT,” “colorectal cancer,” and “colon cancer.” Spectral CT has shown promise in improving CRC detection and T staging accuracy, increasing sensitivity for lesion characterization, and aiding prognostic assessment after chemotherapy using baseline spectral parameters. Early evidence suggests it may also help predict lymph node metastasis and identify patients at risk of early postoperative metastases or surgical complications. Spectral parameters have been correlated with KRAS mutation, Ki-67 index, microsatellite instability, lymphovascular, perineural, and extramural vascular invasion, as well as microvessel density. However, most studies remain small and observational, highlighting the need for validation in larger, multicenter cohorts. Standardization and the time-intensive nature of image segmentation currently limit widespread adoption. Nevertheless, spectral CT is expected to play an increasing role in CRC evaluation by providing quantitative, predictive imaging biomarkers. Integration with artificial intelligence, particularly deep learning and automated segmentation, will likely expand both research and clinical applications.

**Critical relevance statement:**

This article explores the current applications of spectral CT in colorectal cancer by outlining the fundamentals of spectral CT, the spectral parameters used to assess, stage, and predict the prognosis of primary and metastatic disease, as well as the main findings from the current literature.

**Key Points:**

- Spectral CT may be helpful in the detection of colorectal primary tumors, lymph node metastases, and liver metastases, as well as in predicting treatment response.
- Spectral CT offers a non-invasive method to assess genetic mutations and prognostic factors associated with colorectal primaries.
- The lack of standardization in technology and measurement methods limits its applicability in clinical practice.

**Graphical Abstract:**

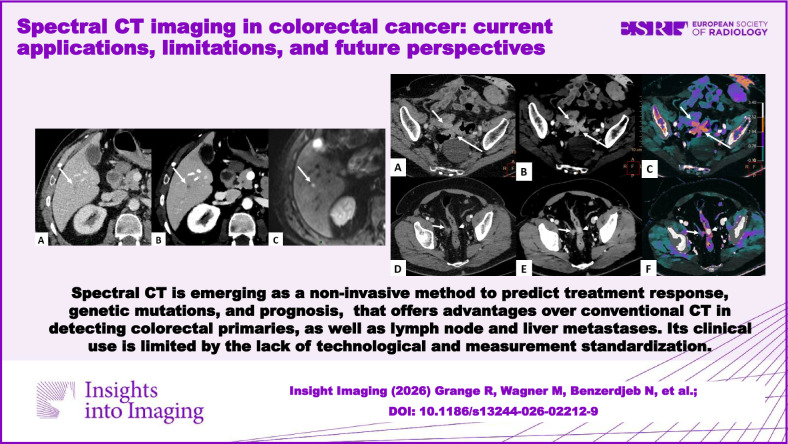

## Background

Colorectal cancer (CRC) is the third most common malignancy worldwide and remains a leading cause of cancer-related mortality [1]. Early detection and accurate staging are crucial for improving patient outcomes, as timely intervention significantly enhances survival rates and enables personalized treatment strategies [2, 3]. Colonoscopy with biopsy remains the gold standard for histopathological diagnosis and mutation detection [4, 5]. Conventional CT provides information on primary tumor localization, TNM staging, local invasion, and distant metastases [6]. However, its sensitivity is relatively low (around 75%) for detecting early primary stages (T1–2) [7]. CT colonography can improve the detection rate for CRC and large polyps, but it has limited sensitivity for detecting small or flat lesions, which account for only a negligible percentage of cancers [8, 9]. Moreover, conventional CT offers limited quantitative information about primary CRC, typically restricted to attenuation values and lesion length [10].

Spectral CT using dual-energy computed tomography (DECT) technology has a broad range of applications, from oncology to cardiovascular imaging, due to its ability to evaluate tissue properties, provide more quantitative data, and improve diagnostic accuracy [11–15]. Unlike conventional CT, which utilizes a single polychromatic energy spectrum, spectral CT analyzes how different tissues interact with X-rays at varying photon energies. DECT is based on the acquisition or detection of two distinct X-ray energy spectra, one at low energy and one at high energy. This dual-energy information allows for the reconstruction of various spectral image types, such as virtual monoenergetic images (VMI). Furthermore, by exploiting differences in material attenuation at both energy levels, material decomposition techniques can be applied to generate iodine overlay maps and quantify iodine concentration (IC), aiding in tissue characterization and perfusion assessment. Several techniques of acquisition are commercially available [16]. The dual-source dual-energy CT (DS-DECT) approach uses two independent X-ray tubes and corresponding detectors to acquire data at different energy levels simultaneously, ensuring true temporal alignment of dual-energy datasets. Another technique, known as rapid kVp switching, uses a single X-ray tube that rapidly alternates between low and high tube voltages during a single gantry rotation, enabling near-simultaneous acquisition of spectral data. Dual-Layer CT (DLCT), on the other hand, employs a single X-ray source and a detector composed of two stacked layers: the upper layer captures predominantly low-energy photons, while the lower layer detects higher-energy photons. This allows continuous spectral acquisition without the need for pre-scan selection (Table 1). Beyond its benefit in reducing artefacts and lowering radiation dose, spectral CT has emerged as a promising technology to improve tumor detectability in various types of cancers, notably by increasing the contrast-to-noise ratio at low energies [17–21].

**Table 1:** Main actual spectral CT systems

Technology type	Operating principle	Acquisition parameter	Manufacturers	Advantages	Limitations
Dual-source dual-energy CT (DS-DECT)	Two X-ray tubes and detectors operate at different energy levels simultaneously.	kVp: 80/140 Simultaneous acquisition Pitch range from 0.2 to 1.2	Siemens Healthineers	High temporal resolution Good energy separation Suitable for cardiac and high-speed imaging	Expensive Requires complex calibration and synchronization
Rapid kVp switching	Single X-ray source rapidly switches between low and high energy during a single rotation	kVP: switch 80/140 kVp every ~0.25 ms Single source Pitch range from 0.531 to 1.531 Gantry speed is limiting factor	GE Healthcare	Fast spectral acquisition No moving parts Good image quality	Temporal resolution limited by switching speed Less suitable for very rapid or irregular motion
Dual-layer CT (DLCT)	A single X-ray source and detector with two layers: top captures low-energy photons, bottom captures high-energy photons.	Tube voltage typically 120 kVp Pitch range from 0.07 to 1.5 Simultaneous acquisition No kVp switching Energy separation via detector	Philips Healthcare	Always on spectral mode No need for pre-scan selection No motion misregistration	Slightly lower energy separation than dual-source Fixed energy bins

*DS-DECT* dual-source dual-energy computed tomography, *DLCT* dual-layer computed tomography

This review aims to summarize the current applications of spectral CT in CRC by outlining the fundamentals of spectral CT, the spectral parameters used to assess primary and metastatic CRC, and the main findings from current literature. Additionally, this review discusses the limitations of spectral CT in CRC and explores potential future directions.

## Methodology

We searched PubMed, Web of Science, and Google Scholar for literature on the application of spectral CT in CRC. The following search terms were used: “spectral CT,” “spectral imaging,” “dual-layer spectral CT,” “dual-energy spectral CT,” “colorectal cancer,” and “colon cancer.” The inclusion criteria were as follows: (1) clinical retrospective or prospective studies focused on CRC; (2) studies examining at least one spectral CT parameter; and (3) articles published in English. Exclusion criteria included: reviews, letters, editorials, comments, case reports, and unpublished articles (unavailable full texts and preprints).

Study selection was based on screening the titles and abstracts according to the prespecified criteria. Articles were excluded if they met at least one of the exclusion criteria or were deemed irrelevant. To minimize the risk of excluding potentially relevant literature, full texts were retrieved and reviewed when eligibility was uncertain.

## Basics of spectral CT

### Spectral parameters used for colorectal evaluation

**Fig. 1:**
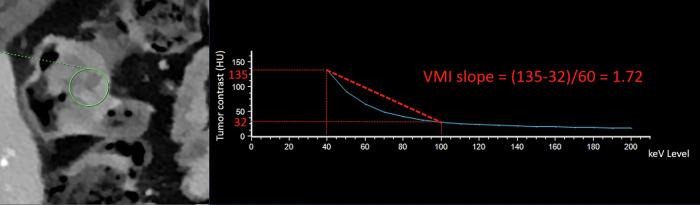
58-year-old male patient with a right pT3N2 right-sided colon cancer. DLCT (Iqon, Philips) in axial section at VP with different energy levels from 40 keV to 200 keV using a single manually outlined ROI. Calculated VMI slope _40-100_ using a single circular ROI was 1.72. CRC, colorectal cancer; VMI, virtual monoenergetic images; ROI, region of interest

Spectral CT provides various quantitative parameters. VMI reconstructs images at specific energy levels (measured in keV), simulating a monochromatic X-ray source. This allows taking benefit of the photoelectric effect at low energy (40–60 keV) to boost the iodine contrast, whereas higher-energy VMI (100–140 keV) reduces beam-hardening artefacts [22]. Notably, a VMI at approximately 70 keV provides attenuation similar to that of a polychromatic image acquired at 120 kVp. To take benefit of the rate of change in attenuation across different monochromatic energy levels, VMI slope is being used for tissue characterization. Tissues with high vascularity, such as aggressive tumors, tend to exhibit a steeper slope, while necrotic or avascular tissues show a flatter slope. More particularly, different values of VMI slopes are available (Fig. 1). For VMI slope _40-100_ and VMI slope _40-90_, the formulas are as follows:

$${{{\rm{VMI\,slope}}}}_{40-100}=({{\rm{VMI}}}\,40{{\rm{keV}}}{{\rm{\hbox{--}}}}{{\rm{VMI}}}\,100{{\rm{keV}}})/60$$

$${{{\rm{VMI\,slope}}}}_{40-90}=({{\rm{VMI}}}\,40{{\rm{keV}}}{{\rm{\hbox{--}}}}{{\rm{VMI}}}\,90{{\rm{keV}}})/50$$

IC measures the absolute amount of iodine (mg/mL) in a tissue region of interest (ROI), providing insights into vascularity. Malignant tumors typically exhibit increased iodine uptake due to higher vascularity, whereas necrotic or fibrotic tissues show lower iodine levels [23]. nIC, defined as the ratio of IC in the tumor to that in the aorta, is calculated as:

$${{\rm{nIC}}}={{\rm{IC\,lesion}}}/{{\rm{IC\; aorta}}}$$

IC in the aorta is typically measured using a unique circular ROI placed in the central 2/3 of the lumen of the aorta. This parameter helps minimize variability, standardize measurements, and enhance the accuracy of tumor characterization [24].

The effective atomic number (Zeff) represents the overall atomic composition of a tissue, which can be derived from spectral CT data. Normalized Zeff (nZeff) has also been used [25, 26] and is defined by the following formula:

$${{\rm{nZeff}}}={{\rm{Zeff\; lesion}}}/{{\rm{Zeff\; aorta}}}$$

Extracellular volume fraction (ECV*f)* quantifies the proportion of extracellular space within a tissue by analyzing iodine uptake, using the following formula:

$${{\rm{ECV}}}f( \% )=(1-{{\rm{hematocrit}}})\,{{\rm{x}}}\,({{{\rm{IC}}}}_{{{\rm{tumor}}}}/{{{\rm{IC}}}}_{{{\rm{aorta}}}}){{\rm{x}}}100$$

This parameter provides information on tissue composition and is particularly useful for differentiating highly cellular tumors from fibrotic or necrotic tissue [27]. ECV*f* has been proven to evaluate cardiac or liver fibrosis and may also assist in distinguishing treatment-induced fibrosis from residual tumor tissue in CRC. Additionally, it may serve as a useful biomarker for predicting tumor behavior and therapy response.

Arterial enhancement fraction (AEF) using IC at arterial phase (AP) and venous phase (VP) has also been studied [28]:

$${{\rm{AEF}}}={{\rm{IC}}}_{{\rm{AP}}}/{{\rm{IC}}}_{{\rm{VP}}}$$

Alternatively, AEF has also been defined using nIC [29]:

$${{\rm{AEF}}}={{\rm{nIC}}}_{{\rm{AP}}}/{{\rm{nIC}}}_{{\rm{VP}}}$$

Venous enhancement fraction (VEF), iodine water ratio (IWR) and normalized IWR (nIWR): IWR_tumor_ / IWR_aorta_ have also been used [30].

### Modality of measurement of spectral parameters in CRC evaluation

**Fig. 2:**
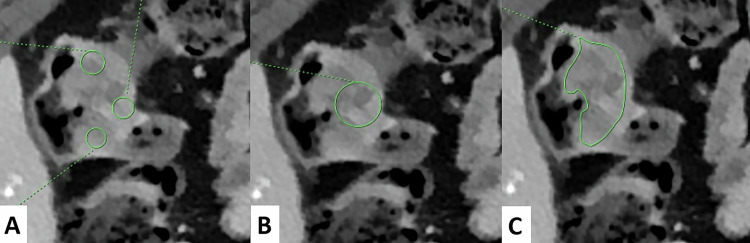
58-year-old male patient with a right pT3N2 right-sided colon cancer. DLCT (Iqon, Philips) in axial section at VP with a 40 keV VMI image. Three different methods of ROI delimitation of the primary tumor are shown: **A** Three circular ROIs, **B** One largest circular ROI, **C** Manually outlined ROI. DLCT, dual-layer computed tomography; ROI, region of interest; VMI, virtual monoenergetic images

**Fig. 3:**
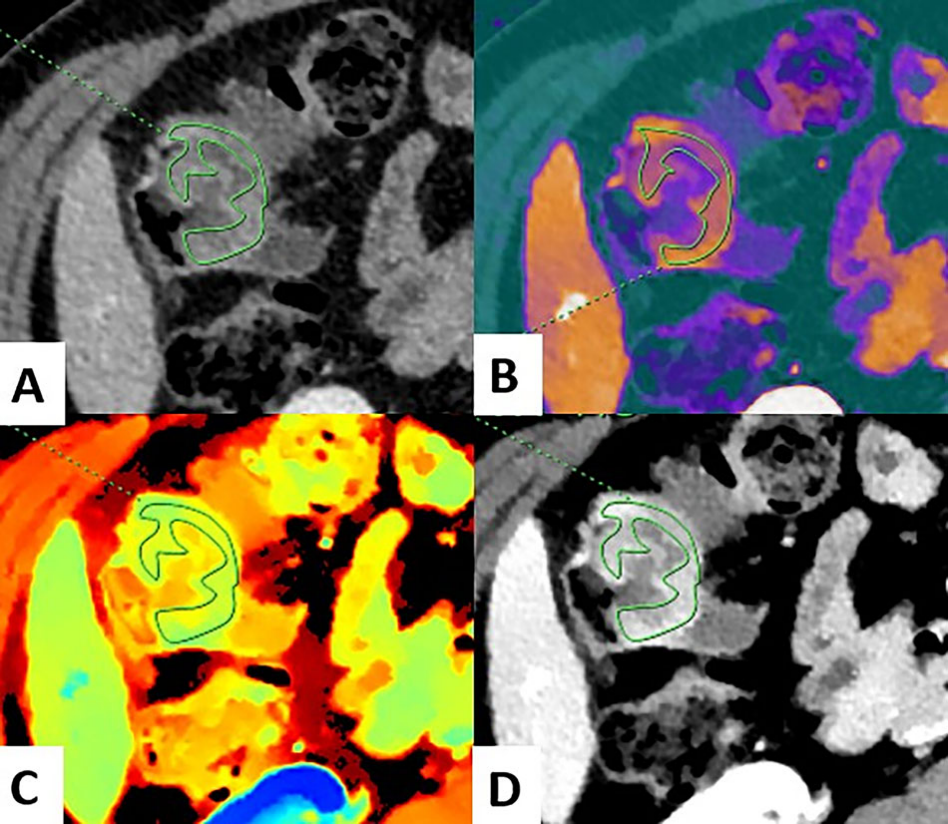
58-year-old male patient with a right pT3N2 right-sided colon cancer. DLCT (Iqon, Philips) in axial section at VP with manually outline ROI. **A** Conventional CT. **B** Iodine map. **C** Z-eff map. **D** 40 keV VMI. CRC, colorectal cancer; ROI, region of interest; VMI, virtual monoenergetic images; VP, venous phase; Zeff, Z-effective

In the evaluation of primary CRC, three main ROI measurement techniques have been described across retrospective studies (Figs. 2 and 3). The most commonly used method involves placing circular ROIs on the largest cross-sectional area, while avoiding areas of necrosis or calcifications. To better capture tumor heterogeneity, multiple small circular ROIs, typically 3, have also been used (Table 2). Another method involves manual delineation along the contour at the level of the largest axial diameter of the tumor [31]. To minimize measurement bias, delineations of the upper and lower slices have also been used [32]. Interobserver agreement was evaluated in Chen et al’s study, reporting an ICC greater than > 0.8 in both AP and VP [33]. Regarding lymph node metastases (LNM) and colorectal liver metastases (CRLM), circular ROI remains the standard measurement technique [26, 30, 34–36]. Typically, three measurements are taken by two experienced radiologists blinded to the studied groups, with the average of these values used as the final recorded value [37, 38].

**Table 2:** Summary of clinical studies studying quantitative spectral parameters in CRC

Organ	Study	No. of patients	Acquisition phases	Method of ROI delimitation	Determination	Most predictive spectral parameters	Associated parameters
Primary colorectal cancer (CRC)	Cao [84]	173	AP, VP	2 radiologists, 1 circular ROI, 3 times	Histologic grade	IC	Age, T stage, N stage
	Gong [83]	81	AP, VP	2 radiologists, 3 circular ROIs at 3 consecutive image levels	Histologic grade	IC, nIC	No
	Chuang-Bo [38]	47	AP, VP	2 radiologists, 3 circular ROIs	Histologic grade	IC, nIC, VMI slope	No
	Chen [42]	131	AP, VP	2 radiologists, manually outline ROI on each slide	T staging, Histologic grade	IC, VMI slope, Zeff	No
	Tan [28]	85	VP	2 radiologists, 1 circular ROI	Complications after surgery	VMI_40_	Visceral fat area
	Arico [37]	66	AP, VP	2 radiologists, 3 circular ROIs	SNR, CNR for T1,2 CRC	VMI_40_	No
	Chen [31]	88	AP, VP	2 radiologists 1 manually outline ROI	Ki-67 HER2	IC, VMI slope, VMI_40_, Zeff	No
	Chen [33]	106	AP, VP	2 radiologists 1 manually outline ROI at 3 consecutive levels	LVI, PNI	VMI_40keV_, VMI_100keV,_ VMI slope, IC, Zeff	No
	Cao [92]	124	AP, VP	2 radiologists, 1 circular ROI at 3 consecutive images levels	KRAS mutation	nIC, VMI slope, Zeff	Pericolorectal fat invasion, ATL/LTL ratio
	Zhao [110]	82	AP, VP	1 radiologist 1 manually outline ROI	MVD	IC, nIC	No
	Zhang [32]	118	AP, VP, DP	2 radiologists 1 circular or elliptic ROI at 3 consecutive image levels	LVI PNI	IC, nIC, VMI slope, ECV*f*	CEA, Histological grades, TN status
	Wu [97]	114	AP, VP, DP	2 radiologists, 1 circular ROI	MSI status	nIC, VMI slope, Zeff	No
	Sun [43]	165	AP, VP, DP	2 radiologists, 1 circular ROI	T staging	ECV*f*	No
	Chen [44]	157	AP, VP	1 radiologist, 1 circular ROIs, 3 times	SNR, CNR for CRC T staging	VMI_40keV_, VMI_50keV_	No
	Gao [103]	36	AP, VP, DP	2 radiologists, 1 ROI 3 times	EMVI in rectal cancer	IC, nIC	No
	Lu [127]	62	UN, VP	2 radiologists, manually outline	PNI in rectal cancer	VMI 40	N status
	Chen [35]	82	AP, VP	1 radiologist, 1 ROI 3 times of LN	LNM	nIC	No
Lymph node metastases (LNM)	Qiu [30]	71	AP, VP	1 radiologist, 1 ROI 3 times of LN	LNM	VMI slope, IC, IWR	No
	Yang [26]	178	AP, VP, EP	2 radiologists, manually outline of LN	LNM	nZeff, nIC, VMI slope	Short axis
	Cao [36]	167	AP, VP	2 radiologists, 1 circular ROI of LN	LNM	IC, Zeff	Pericolorectal fat invasion, CEA, CA19-9
	Liu [34]	42	VP	2 radiologists, 1 circular ROI at 3 consecutive sections in LN	LNM	Zeff	Short axis diameter
	Sato [52]	44	AP, VP	1 surgeon, 1 medical student1 circular ROI	LNM in rectal cancer	nIC	Short diameter
	Li [67]	104	VP	4 radiologists, 1 circular ROI of CRLM	CRLM. OS after Folfoxiri + Bevacizumab	IC	Synchronous vs. Metachronous Largest diameter at baseline CEA KRAS mutation
Colorectal liver metastases (CRLM)	Li [68]	88	UN,AP, VP, EP	3 radiologists, 1 circular or oval ROI of CRLM	CRLM. OS after 2 cycles of chemotherapy, including Bevacizumab	ECV*f*	No
	Peng [29]	222	AP, VP, EP	2 radiologists, 1 circular or elliptic ROI of primary CRC	EDM	VEF, 1/nIC, VMI slope	CEA, EMVI
	Lenga [61]	98	VP	2 radiologists, 2 circular ROI of CRLM	CRLM: quantitative size measurements and diagnostic accuracy	VMI_40_	No
	Lenga [62]	53	VP	1 radiologist, 2 circular ROI of CRLM	CRLM: SNR, CNR, image quality, lesion delineation, and image noise	VMI_40_	No
	Bae [63]	173	VP	1 radiologist, 3 circular ROI of CRLM	CNR, image noise, image contrast, lesion conspicuity, lesion detection, CRLM diagnosis	VMI_50_	No

*AP* arterial phase, *ATL/LTL* axial tumor length/longitudinal tumor length, *CA* carbohydrate antigen, *CAE* carcinoembryonic antigen, *CRC* colorectal cancer, *CRLM* colorectal liver metastases, *CT* computed tomography, *DLCT* dual-layer computed tomography, *DP* delayed phase, *ECVf* extracellular volume fraction, *EDM* early distant metastasis, *HER* human epidermal growth factor receptor, *IC* iodine concentration, *IWR* iodine water ratio, *Ki-67* antigen Kiel 67, *LN* lymph node, *LVI* lymphovascular invasion, *MVD* microvessel density, *nIC* normalized iodine concentration, *nZeff* normalized Z-effective, *ROI* region of interest, *VMI* virtual monoenergetic images, *VP* venous phase, *Zeff* Z-effective, *UN* unenhanced CT, *VEF* venous enhancement fraction

## Applications of spectral CT in CRC

### Early detection and diagnosis of primary

**Fig. 4:**
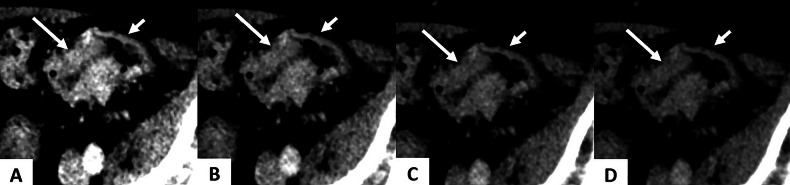
54-year-old male with pT3 sigmoid adenocarcinoma. DS-DECT (Siemens Force in axial section at VP with VMI at different energy levels. **A** 40 keV, **B** 50 keV, **C** 60 keV, **D** 70 keV. 40 keV VMI image shows improved contrast between high enhancement of the tumor (long arrow) and adjacent enhancement of normal bowel (short arrow). DS-DECT, dual-source dual-energy computed tomography; ROI, region of interest; VMI, virtual monoenergetic images; VP, venous phase

**Fig. 5:**
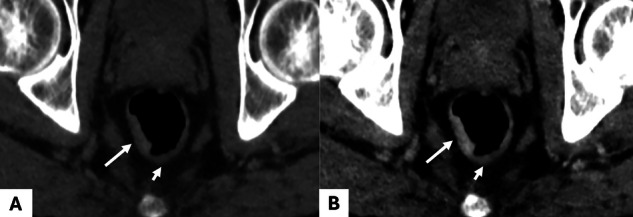
57-year-old man with T2 low rectal cancer. DS-DECT (Siemens Force) in axial section at VP. **A** 70 keV VMI image shows minimal attenuation difference between the tumor (long arrow) and the adjacent normal rectal wall (short arrow). **B** 40 keV VMI image shows increased contrast between the tumor (long arrow) and the adjacent normal rectal wall, increasing contrast-noise-ratio between the tumor and the adjacent rectal wall. DS-DECT, dual-source dual-energy computed tomography; VMI, virtual monoenergetic images

Early detection of small T1-2 CRC via CT scan is a major challenge as the contrast between tumors and surrounding normal colon tissue is low [39]. A retrospective analysis of 153 CRC cases reported a 70.2% sensitivity and 79.2% specificity for detecting T1–T2 stages using CT [7]. By increasing the contrast between tumor and adjacent bowel at low energy, VMI could enhance CRC detection compared to conventional CT. Arico et al evaluated both qualitative and quantitative spectral parameters in 66 patients with biopsy-proven CRC. Signal-to-noise ratio (SNR) and contrast-to-noise ratio (CNR) were calculated across different VMI reconstructions at late AP. Their findings demonstrated that both SNR and CNR were significantly higher at VMI_40_ compared to other VMI reconstructions (*p* < 0.05) (Figs. 4 and 5). In the qualitative assessment, the addition of VMI_40_ to conventional CT images significantly improved the diagnostic accuracy, with a higher area under the curve (AUC) for CRC detection for both readers (*p* < 0.05) [37]. Interestingly, the improvement was greater in the less experienced radiologist.

### T staging of CRC

**Fig. 6:**
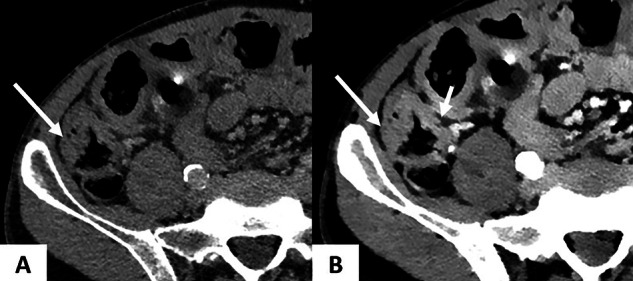
A 53-year-old woman with a sigmoid adenocarcinoma treated with four cycles of FOLFOX. DS-DECT (Siemens Force) in axial section at VP. **A** The conventional CT image shows right colon wall thickening (arrow). **B** The 40 keV VMI image shows greater attenuation of the primary tumor with clear involvement of pericolic fat (short arrow). Histopathology of the surgical specimen confirmed a pT3 adenocarcinoma due to invasion of the pericolic fat. CT, computed tomography; VMI, virtual monoenergetic images; VP, venous phase

Accurate T staging using conventional CT remains particularly challenging and may lead to mis-staging, particularly in patients with ascending or transverse colon or low visceral fat tissue [40, 41]. Compared with pT, the reference standard, preoperative CT was associated with up to 39% of mis-staging [40]. This is clinically significant, as decisions regarding neoadjuvant chemotherapy (NAC), more frequently used in advanced stages, rely heavily on accurate staging to guide its indication. Chen et al evaluated the ability of quantitative parameters of preoperative DLCT to predict T stage and histological grade in 131 CRC patients (Fig. 6). Zeff, IC, and VMI slope of T3/T4 stage CRC were significantly higher than those of T1/T2 [42]. In a prospective multicentric study of 166 CRC lesions, Sun et al reported that all spectral parameters, such as VMI, IC, and Zeff, were higher in pT3 stage than in pT1-T2 [43]. Notably, ECV*f* demonstrated excellent performance in predicting pT staging in CRC, with an AUC of 0.919 and 0.892 in training and external validation groups [43]. Recently, Chen et al found that 40–50 keV VMIs derived from DLCT significantly enhanced image quality in preoperative T staging of CRC. Moreover, 40–50 keV VMIs outperformed conventional CT in the accurate delineation of early-stage tumors (T1-T2) [44].

### Diagnosis of lymph node metastasis (LNM)

LNM and LN ratio are associated with a poorer OS [45]. However, predicting LNM in CRC remains challenging with conventional CT. Generally, a short axis > 1 cm is considered suspicious for metastases. This cutoff does not allow systematic detection of micrometastases. In fact, only 50% of patients with LN larger than 1 cm are pathologically metastatic. Additionally, inflammatory or infectious intercurrent disease during preoperative chemotherapy can lead to false positives [46–48]. Morphologic features, including irregular border, heterogeneity and round shape, or aggregation in cluster, have limited accuracy [49].

**Fig. 7:**
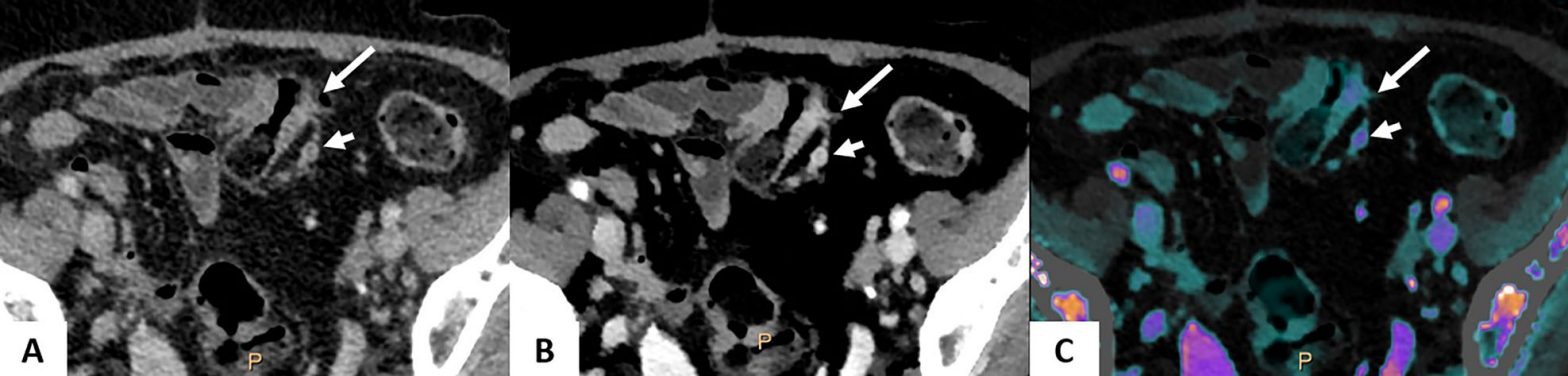
A 61-year-old woman with sigmoid cancer and synchronous peritoneal metastases from a sigmoid adenocarcinoma, treated with four cycles of FOLFOX. DS-DECT (Siemens Force) in axial section at VP. **A** The conventional CT image shows sigmoid wall thickening (long arrow) and an adjacent LN (short arrow). **B** The 40 keV VMI image allows better delineation of the tumor, showing tumoral infiltration of the mesosigmoid fat (long arrow) and marked enhancement of the adjacent LN (short arrow). **C** The iodine map image confirms significant iodine uptake within the primary tumor (2.09 mg/mL) and the LN (2.31 mg/mL). Histopathology confirmed a T3N1 tumor. CT, computed tomography; VMI, virtual monoenergetic images; VP, venous phase

A nationwide registry-based study including 4849 cT1-2 CRC reported low diagnostic accuracy (67%) and sensitivity (26%) of conventional CT in detecting LNM from CRC [50]. This performance is even lower in CRC cases with microsatellite instability (MSI), where over-staging is common [49, 51, 52]. Chen et al evaluated the utility of preoperative spectral CT in detecting LNM from 82 CRC patients [35]. The study included 82 patients with proven CRC, divided based on their metastatic status. Single-energy CT values at 40–140 keV, as well as IC, nIC, VMI slope and Zeff were higher in the non-metastatic group compared to the metastatic group (*p* < 0.05). At AP, the AUC for nIC in determining LN status was 0.873. Cao et al developed a nomogram including clinical factors and DECT parameters of the primary tumor to predict LNM in 167 patients with pathologically confirmed CRC [36]. The authors found that IC at AP and VP, and Zeff were independent predictors for LNM. The nomogram including these spectral parameters, pericolorectal fat invasion, CEA and CA19.9 achieved an AUC of 0.876 in the training cohort and 0.852 in the validation cohort. Liu et al demonstrated that the inclusion of spectral parameters such as Zeff, combined with the short-axis diameter, enhances precision on diagnostic accuracy of LN in pT1-2 rectal cancer (RC) [34]. In a prospective study of 71 patients with CRC, they also reported the ability of spectral CT to distinguish LNM+ and LNM-. LNM+ showed higher values for VMI slope, IC and IWR at AP and VP (*p* < 0.05) [30] (Fig. 7). Yang et al investigated the largest LN in 178 patients with CRC (72 LNM+ and 106 LNM-). In this study, all DECT parameters were significantly lower in LNM+ . The authors also reported that nZeff, nIC and VMI slope significantly outperformed morphologic criteria in predicting LNM+ [26]. Finally, Sato et al assessed the ability of DECT to predict pararectal and lateral pelvic LNM in 44 patients with RC. nIC was significantly lower in pararectal LNM at AP and VP, and in lateral pelvic LNM at VP [53]. Thus, these studies report contradictory findings, with LNM+ showing either higher or lower spectral parameters depending on the study [26, 30, 53]. LN enhancement may be influenced by several factors, including immune infiltration, the effects of immunotherapy, and tumor involvement. These parameters, along with hemodynamic conditions, can alter LN vascularity and permeability of LN, explaining such results.

### Colorectal liver metastasis (CRLM)

#### Detection

**Fig. 8:**
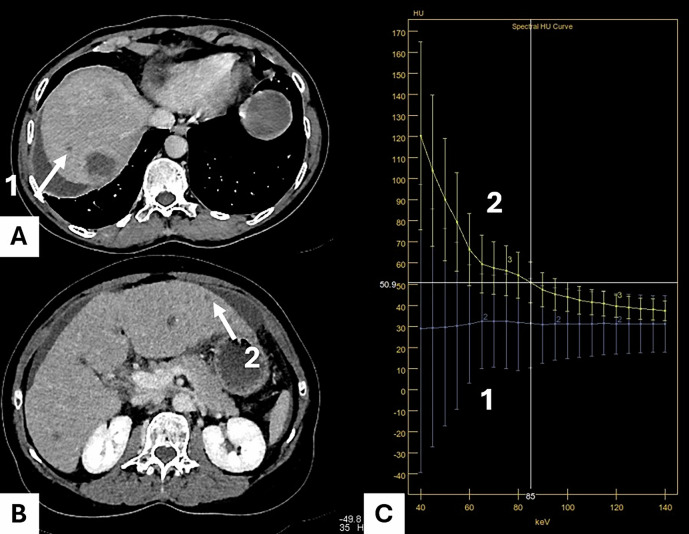
80-year-old female with recent discovery of T3N2 right colon cancer and liver metastases. Rapid kVp switching CT (Frontier GE) in axial section at VP. **A** The 55 keV VMI image shows a 4-mm hypodense lesion in the hepatic dome (arrow, lesion 1). **B** The 55 keV VMI image shows a 6-mm mostly hypodense lesion in hepatic segment III (arrow, lesion 2), located below. **C** On spectral attenuation curves, lesion 1 demonstrates a flat profile, consistent with a liver cyst (VMI slope = 0). Lesion 2 demonstrates spectral decay with a VMI slope 40–100 = (120–45)/60 = 1.25. MRI confirmed the cystic nature of the dome lesion and the solid nature of the segment III lesion. CT, computed tomography; VMI, virtual monoenergetic images

**Fig. 9:**
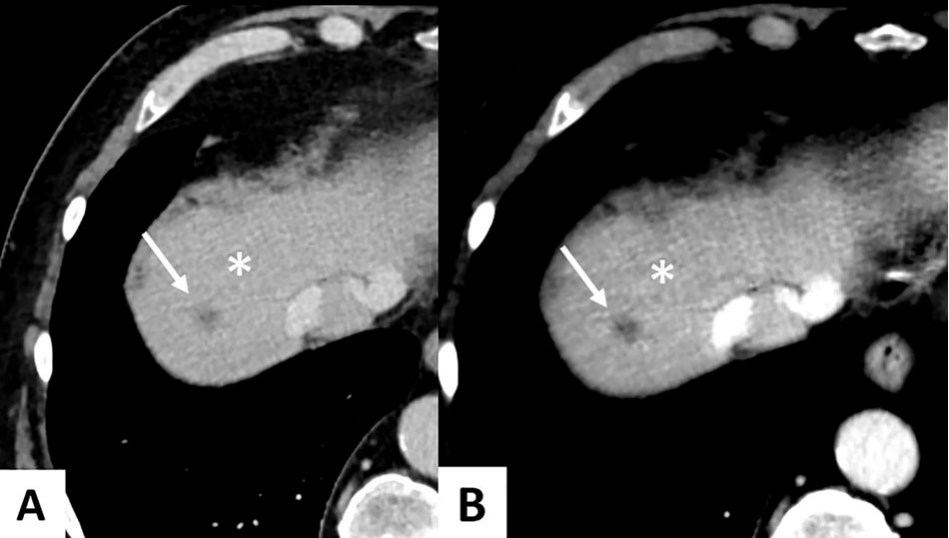
81-year-old patient with a recent diagnosis of right-sided colon adenocarcinoma and a single liver metastasis at baseline evaluation. DS-DECT (Siemens Force) in axial section at VP. **A** The conventional CT image shows a poorly defined 11 mm lesion (arrow) in the hepatic dome (star). **B** The 40 keV VMI image improves the contrast between the lesion (arrow) and the adjacent liver tissue (asterisk). Percutaneous biopsy of the lesion under CT guidance confirmed the metastatic nature of the lesion. CT, computed tomography; DS-DECT, dual-source dual-energy computed tomography; VMI, virtual monoenergetic images

CRLM typically appear as hypoattenuating lesions on contrast-enhanced CT during VP [54]. While conventional CT remains a valuable imaging modality for the detection of CRLM, its sensitivity for lesions < 1 cm remains suboptimal—only around 43% [55, 56]. Moreover, liver MRI has demonstrated superior accuracy for CRLM detection, and should be the gold standard for preoperative evaluation, even following NAC [56–58]. This limited sensitivity for small lesions is due to reduced contrast resolution between tumor and liver. Spectral CT at low energy (40–50 keV) increases the contrast between hypoattenuating LM and surrounding liver parenchyma by amplifying iodine attenuation [59]. In this context, Lenga et al conducted a comparative study in 98 CRC patients (46 with CRLM), evaluating DECT VMI against standard reconstructions [60]. Inter-rater agreement of lesion size was higher for VMI_40_ (ICC = 0.88). Both sensitivity and diagnostic accuracy for the detection were significantly higher for VMI_40_ compared to standard reconstructions (90.6% vs. 80.6% and 89.1% and 81.3% respectively, *p* < 0.001). The same authors evaluated the effectiveness of the noise-optimized VMI technique in assessing hypoattenuating CRLM using abdominal DECT in 53 patients with histologically confirmed CRLM. VMI_40_ demonstrated the highest SNR and CNR [61]. Regarding qualitative analysis, VMI_60_ provided the best overall image quality ratings, while VMI_40_ provided superior lesion delineation (*p* ≤ 0.001). The authors concluded that low-energy reconstructions significantly enhance image quality and lesion delineation in patients with CRLM (Figs. 8 and 9, Supplementary 1). Recently, Bae et al evaluated the potential of VMI in assessing CRLM in 173 patients with 797 focal liver lesions, including 463 CRLM. The authors concluded that the VMI_50_ improved lesion detection but not CRLM diagnosis [62].

#### Overall survival and response after chemotherapy

The evaluation of chemotherapy response is primarily determined by size-based criteria, using RECIST guidelines [63]. However, RECIST criteria may be suboptimal for the early assessment of efficacy, particularly for agents like Bevacizumab, which operates through a cytostatic mechanism [64]. Moreover, CT attenuation of CRLM before targeted therapy has been proven to be a prognostic factor of prolonged overall survival (OS) and better tumor response [65]. Li et al retrospectively analyzed 104 patients with CRLM treated with Folfoxiri and Bevacizumab, using spectral CT parameters at VP. Higher baseline IC in target lesions was found to be an independent risk factor for poor OS, along with baseline target diameter, CEA, Kirsten rat sarcoma (KRAS) mutation and metachronous liver metastasis. Moreover, IC > 4.75 (100 µg/cm^3^) demonstrated a good performance to predict poor responders, with an AUC = 0.916, and a sensitivity and specificity of 80.3% and 96.4%, respectively [66]. The same team reported that the relative change in ECV*f* after 2 cycles of chemotherapy combined with Bevacizumab was an independent predictor for tumoral response [67]. Conversely, Huellner et al assessed, in a study involving 55 CRC patients with synchronous LM, the prognostic value of CT parameters at baseline to predict time to progression or OS, and no predictive parameters were found [68]. Overall, while several studies highlight the promising potential of spectral CT–derived parameters to refine the prediction of therapeutic response, the evidence remains heterogeneous, underscoring the need for larger prospective studies to confirm the true clinical value of these biomarkers.

### Diagnosis of peritoneal metastases (PM)

**Fig. 10:**
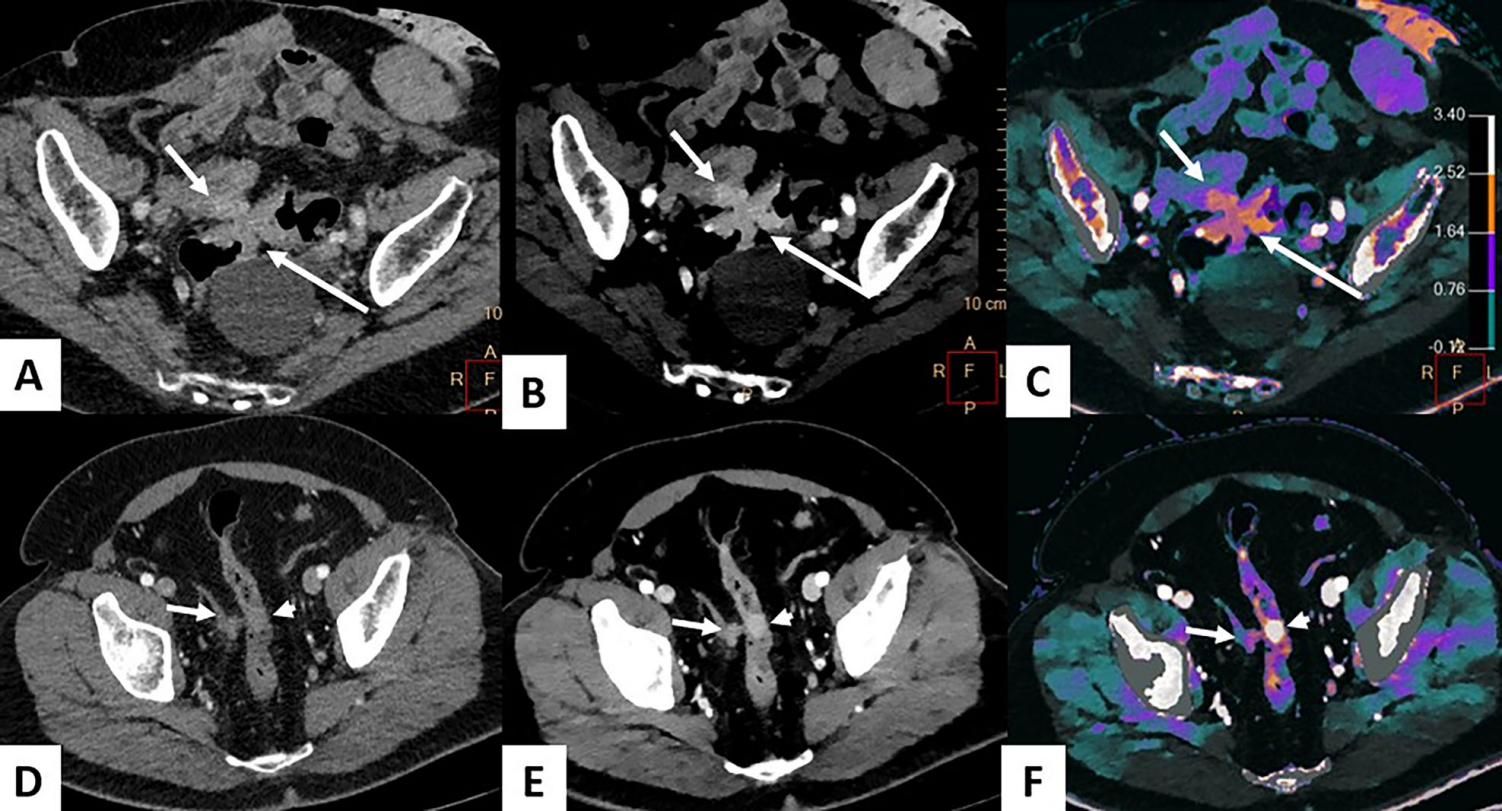
65-year-old female with synchronous PM from a sigmoid primary tumor. DLCT (Iqon, Philips) in axial section at VP, performed after NAC. **A** The conventional CT image shows an irregular circumferential parietal thickening of the primary sigmoid lesion (long arrow), while the nodule in contact with the small-bowel serosa is difficult to distinguish (short arrow). **B** The 40 keV VMI image provides better delineation of both the nodule and the primary tumor. **C** The iodine map image confirms significant iodine uptake in both the primary tumor and the peritoneal nodule. **D** The conventional CT image also reveals a 13 mm nodule in the right iliac fossa, located against the parietal peritoneum (arrow), while the nodule adjacent to the serosa of the distal sigmoid is poorly visualized (arrowhead). **E** The 40 keV VMI image enhances the contrast between the peritoneal nodule (arrow), the sigmoid serosal nodule (arrowhead), and the adjacent structures. **F** The iodine map image shows significant iodine uptake in the parietal peritoneal nodule (short arrow), and especially in the nodule of the sigmoid serosa (arrowhead). CRS, cytoreductive surgery; CT, computed tomography; DLCT, dual-layer computed tomography; NAC, neoadjuvant chemotherapy; PM, peritoneal metastases

**Fig. 11:**
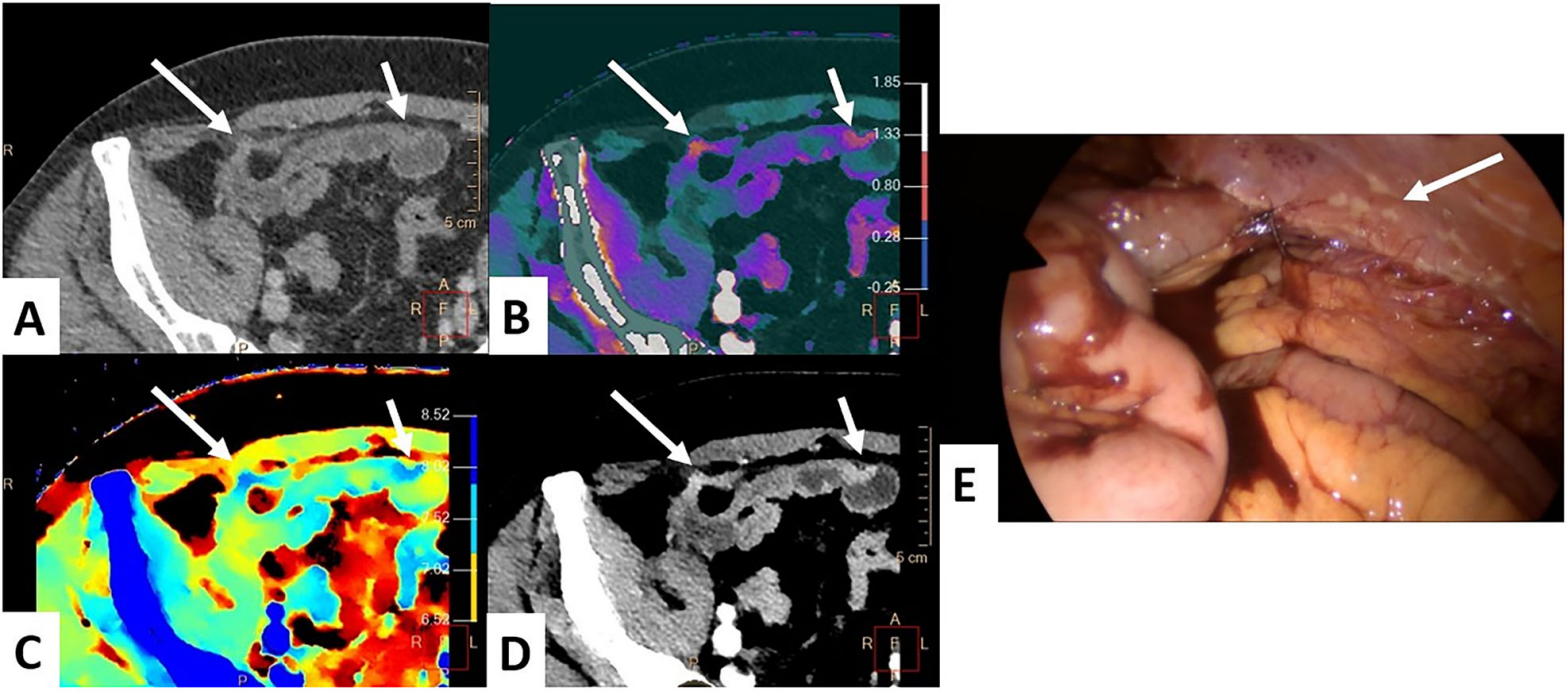
61-year-old man scheduled for CRS and HIPEC for a transverse colon adenocarcinoma with synchronous PM. DLCT (Iqon, Philips) in axial section at VP. **A** The conventional CT image shows a barely visible nodular thickening of the parietal peritoneum in the right iliac fossa (long arrow) and the small-bowel serosal nodule (short arrow). **B**–**D** The iodine map (**B**), Zeff map (**C**), and 40-keV VMI image (**D**) show much better the detection of the nodular thickening (long arrow), compared with the adjacent abdominal wall and small-bowel loops, as well as the small-bowel serosal nodule (short arrow). **E** Laparoscopy confirms both peritoneal metastases, including the parietal peritoneal nodule (long arrow). CRS, cytoreductive surgery; CT, computed tomography; DLCT, dual-layer computed tomography; HIPEC, hyperthermic intraperitoneal chemotherapy; PM, peritoneal metastases; Zeff, Z-effective

PM from CRC are associated with poor prognosis [69, 70]. In selected patients, achieving a complete cytoreductive surgery (CRS), potentially combined with hyperthermic intraperitoneal chemotherapy (HIPEC), significantly improved OS [71]. CT scan is the preferred imaging modality for the detection of PM, due to its high availability, routine use in oncology and high spatial resolution. However, CT tends to underestimate the extent of small PM, due to the low contrast between peritoneal lesions and the serosal surfaces [72–74]. Spectral CT, through enhanced contrast at low energy levels, offers a promising approach for improving lesion detection (Figs. 10 and 11, Supplementary 2). Additionally, IC provides valuable insights into the tissue characteristics of lesions [75]. However, the role of spectral CT in detecting PM regarding CRC primaries remains unstudied. Nevertheless, Lennartz et al assessed the value of spectral CT in differentiating between benign from malignant peritoneal lesions in a cohort of 30 patients with PM and 30 patients with benign peritoneal lesions. Compared with conventional imaging, the combination of conventional imaging and iodine overlay improved specificity in the assessment of PM at comparable sensitivity, particularly in postoperative patients [75]. Although the lack of a large study, this application of spectral imaging seems promising, as the underdiagnosis of peritoneal lesions on CT is primarily related to insufficient contrast resolution [76]. The combination of CT’s wide availability and high spatial resolution, with the enhanced contrast at low-energy levels and iodine mapping, is expected to improve the sensitivity for detecting peritoneal metastases.

### Predictive factors after colorectal surgery

#### Prediction of complications after colorectal surgery

Delayed initiation of adjuvant chemotherapy may adversely impact OS after CRC surgery [77]. Tan et al investigated the predictive value of spectral parameters of primary CRC in identifying surgical complications after CRC surgery by comparing 27 patients who developed postoperative complications and 58 patients who did not [28]. VMI_40_ at VP and visceral fat area were found to be independent predictors of postoperative complications. A model combining VMI_40_ of primary and visceral fat area yielded an AUC of 0.84 with a sensitivity of 77.8% and a specificity of 87.9%. Nevertheless, the study lacks essential information on postoperative complications, as neither their type nor their severity grade is detailed, making it difficult to interpret the clinical significance of the outcomes. Finally, although the combined predictive model appears promising, the absence of external validation limits its applicability in routine practice.

#### Prediction of early distant metastases (EDM) after surgery

Curative surgical resection is the gold standard treatment for non-metastatic CRC [78, 79]. CT is the preferred imaging modality for colon cancer staging and plays an important role in the management of RC patients undergoing therapy. Despite curative intent, approximately 4–6% of patients with CRC will develop EDM (< 6 months) after surgery of primary [29, 80]. Poor tumor differentiation, involvement of mesorectal fascia, and extravascular vascular invasion (EMVI+) have been found to be associated with EDM [80]. However, conventional CT remains limited in predicting EDM. Peng et al evaluated the potential of spectral parameters in predicting early postoperative distant metastases in 222 patients [29]. They found that VEF, VMI slope at VP, and 1/nIC at VP in the primary tumor were significantly different between patients with distant metastases and those without (*p* < 0.05). When combined with CEA levels and EMVI on CT, these parameters further enhanced the predictive performance of both clinical and spectral models, achieving an AUC of 0.887, which was significantly higher than that of the clinical model alone. The study is limited by the very small number of patients who developed EDM. Some of the spectral biomarkers included, such as VEF and 1/nIC, are rarely used or validated in previous DECT literature, which raises concerns about their physiological relevance. Finally, several key parameters related to tumor biology or perfusion showed no association, yet the discussion remains speculative regarding their physiological significance.

### Histopathological and molecular applications

#### Tumor grading

**Fig. 12:**
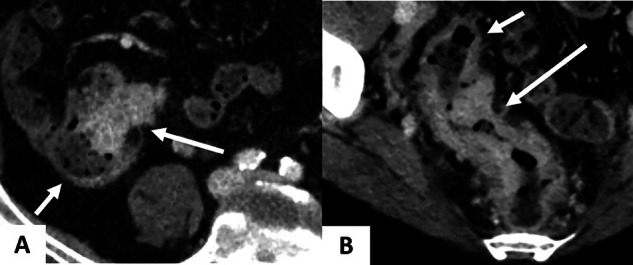
72-year-old (**A**) and 67-year-old (**B**) males with biopsy-proven adenocarcinoma. DS-DECT (Siemens Force) in axial section at VP. **A** The 40 keV VMI image shows a marked enhancement of the primary tumor (long arrow) compared with the adjacent normal wall. IC was 2.1 mg/mL. **B** The 40 keV VMI image also shows a slightly lower enhancement of the primary tumor (long arrow) compared to the adjacent normal wall (short arrow). IC was 1.6 mg/mL. **A** corresponds to well-differentiated tumors, whereas **B** corresponds to a poorly differentiated tumor. DS-DECT, dual-source dual-energy CT; IC, iodine concentration; VMI, virtual monoenergetic images

Tumor grading is correlated to tumor aggressiveness, neoangiogenesis, and prognosis [81, 82]. Well and moderately differentiated (Grade 1 and 2) are considered low-grade, whereas poorly differentiated and undifferentiated (Grade 3 and 4) are considered high-grade. Accurate grading is relevant for surgical planning, treatment strategy and prognosis stratification. Spectral CT has emerged as a non-invasive method for determining tumor grading. Gong et al included 81 patients treated surgically for CRC: a low-grade cancer group (37 patients) and a high-grade cancer group (44 patients) [83]. IC and nIC were significantly higher in high-grade differentiation. Using a threshold value of 1.92 for nIC at AP provided a sensitivity of 70.3% and a specificity of 97.7% with an AUC of 0.95. Chuang-Bo et al included 47 pathologically confirmed CRC. IC, nIC, and VMI slope were significantly higher in the well-differentiated group (*p* < 0.05) (Fig. 12) [38]. Using an IC threshold of 1.13 mg/mL in AP yielded a sensitivity of 81.8% and a specificity of 71.4% significantly superior to using VMI at 70 keV. Cao et al included 173 patients with high-grade (*n* = 65) and low-grade (*n* = 108) CRC, and developed a nomogram to distinguish the two grades [84]. A quantitative nomogram based on age, T stage, N stage and IC value at AP and VP showed excellent performance with an AUC of 0.886 for differentiating between low- and high-grade CRC.

#### Lymphovascular (LVI) and perineural invasion (PNI)

LVI and PNI are significant prognostic markers in CRC, correlating with increased recurrence and reduced survival [85–87]. Accurate preoperative prediction of LVI and PNI status in CRC can be helpful in selecting patients requiring appropriate adjuvant therapy and evaluating prognosis. Zhang et al investigated the value of combining spectral parameters for preoperative evaluation of LVI and PNI in CRC [32]. IC, nIC and VMI slope at VP and DP, along with ECV*f* at DP were significantly higher in the LVI/PNI + group (*p* < 0.05). These parameters, combined with clinical model including CEA, histological grade, TN status, reached the highest diagnostic accuracy, with AUC being 0.857. Chen et al found in a cohort of 106 patients with CRC that spectral parameters such as VMI_40_, VMI_100_, VMI slope, Zeff and IC at VP were significantly higher in patients with LVI [33]. However, no statistical difference was observed with respect to PNI status in this study.

#### KRAS mutation

KRAS is one of the most commonly mutated oncogenes in CRC, and it is associated with poorer DFS and OS in microsatellite-stable (MSS) patients [88–91]. Cao et al developed a predictive nomogram to assess KRAS mutation status in 144 CRC patients, including a 50 KRAS + CRC group and a 74 KRAS- CRC group. VMI slope and Zeff at AP, and nIC at VP were independent predictors for KRAS mutation [92]. These parameters, along with axial-to-longitudinal tumor length ratio and pericolorectal fat invasion, had excellent performance in predicting KRAS mutation, with an AUC of 0.848.

#### Ki-67 and Her-2 expression

Antigen Kiel 67 (Ki-67) is a marker of tumor cell proliferation in CRC. A high Ki-67 index is associated with poor prognosis, higher tumor grade, and increased risk of recurrence after surgery [93–95]. Chen et al evaluated the value of quantitative spectral parameters in DLCT for evaluating the expression of Ki-67 and Her-2 in 88 CRC. The authors found that Ki-67 expression was positively correlated with VMI_40_, Zeff, IC, and VMI slope at VP. The authors showed no correlation between Her-2 expression and DLCT parameters (*p* > 0.05) [31].

#### Microsatellite instability (MSI)

Determining MSI status is critical in guiding CRC treatment strategies, particularly for identifying candidates for immunotherapy before surgery [96]. While colonoscopic biopsy remains the standard method to obtain CRC tissues, its diagnostic utility can be limited due to tumor heterogeneity and colonoscopy sampling limitations. Wu et al evaluated 38 patients with MSI primary and 76 patients with MSS primary. The authors found that the VMI slope at AP was the best parameter to distinguish MSI and MSS primary, with an AUC of 0.803. Combined parameters, including nIC, Z-eff and VMI slope, significantly improved diagnostic ability, with an AUC of 0.886 (sensitivity, 81.6%; specificity, 81.6%) [97].

#### Extramural vascular invasion (EMVI) in rectal cancer

EMVI + RCs are associated with distant metastases, poor prognosis, and EDM after surgery [98–100]. Rectal MRI is the gold standard for evaluation of RC [101, 102]. Nevertheless, this modality is subject to artefacts, time- and cost-consuming. Gao et al evaluated in 36 patients the ability of spectral CT parameters to predict EMVI + patients in RC. EMVI + RC (*n* = 13) exhibited significantly higher IC at the VP and DP [103]. Moreover, nIC at AP, VP and DP were significantly higher in EMVI + RC. nIC at DP showed good performance to distinguish EMVI + RC, with an AUC of 0.983.

#### Microvessel density (MVD)

MVD is a crucial biomarker of tumor angiogenesis, reflecting the extent of vascular proliferation within tumors. Elevated MVD values indicate increased blood vessel formation, which supplies tumors with oxygen and nutrients, thereby facilitating growth, invasion, and metastasis [104–106]. Moreover, high MVD in CRC is associated with poorly differentiated, vascular invasion and poor prognosis [107, 108]. Elevated MVD is also a predictor of recurrence, as highly vascularized tumors tend to exhibit increased resistance to treatment, promoting both local lymphatic spread and distant metastases [109]. As VMI and IC reflect parts of tumoral neoangiogenesis, these parameters could be interesting predictors of tumoral MVD. Zhao et al evaluated the performance of spectral parameters in assessing MVD in 82 patients with colonoscopy or surgically proven CRC (52 low MVD and 30 high MVD). The authors found that AUC for IC, nIC and IC + nIC at AP were all > 0.9, indicating that both single and combined parameters of spectral CT were highly effective in predicting the level of MVD [110].

The histopathological and molecular applications offer an innovative, quantitative, non-invasive approach. However, the current performance of spectral imaging does not yet allow for a sufficiently reliable analysis to replace histological examination. In addition, several correlations, such as those involving KRAS or BRAF mutations, MSI status, and Ki-67 expression, lack a convincing pathophysiological substrate to explain potential variations in spectral parameters.

## Current limitations

Spectral CT imaging offers a wide range of potential applications in CRC. However, several limitations need to be acknowledged.

First, most of previously cited studies are single-center retrospective studies, with relatively small sample sizes, limiting the generalizability of their findings. Large-scale, multicentre, prospective studies with external validations are needed to validate the results of the current literature. Studies reporting correlations (histopathological or molecular applications) are also scarce. Although ROC analyses often yield promising areas under the curve, these studies rarely include external validation cohorts, which substantially limits the generalizability of their findings. Moreover, the reported correlations between mutation profiles and spectral CT parameters frequently lack a robust physiopathological rationale and demonstrate inconsistent reproducibility across studies. Consequently, despite encouraging preliminary results, spectral–molecular associations remain largely exploratory, with insufficient evidence to justify their incorporation into routine clinical decision-making.

Second, at present, spectral CT technology is not widely available. This limited accessibility represents a significant barrier to the design and implementation of large multicenter trials, as well as to longitudinal studies assessing patients before and after treatment. Broader dissemination of spectral CT systems is therefore essential to validate current findings, ensure reproducibility across different clinical settings, and fully establish their role in the management of CRC, including survival data.

Lack of standardized spectral CT protocols further complicates direct comparisons between DECT studies. Differences in scanner technology, energy thresholds, reconstruction algorithms, and post-processing techniques may contribute to inconsistencies across studies [16]. Regarding CRC, the triphasic protocol has been used. However, the inclusion of an unenhanced acquisition and AP is debatable, as it leads to increased radiation exposure for patients, without a clear benefit in reading. The definition of the VMI slope varies across studies (40–90 keV, 40–100 keV, or 40–70 keV), which limits generalizability [31, 38, 42, 92]. The method for measuring ROIs is not standardized, and no quality control for ROI measurement is currently available. Additionally, ROI measurement is time-consuming, and no effective automated method for delineating the primary tumor has been reported in these studies.

Third, CRC presents with diverse histological subtypes and molecular profiles. Spectral CT parameters may not perform equally across all CRC subtypes. This variability complicates the establishment of universal diagnostic thresholds. Notably, for mucinous CRC, a subtype characterized by poor vascularization, which is underrepresented in retrospective studies [111]. Additionally, most existing research has focused on early-stage or locally advanced CRC, while the predictive value of spectral CT in metastatic CRC remains underexplored in the current literature. Findings derived from early-stage CRC may not be applicable to advanced disease, as metastatic lesions often display varying degrees of differentiation and may harbor distinct genetic mutations that contribute to chemotherapy resistance. Moreover, most of the previously cited studies have been conducted in Asian populations, predominantly Chinese cohorts, and are not fully applicable to a Caucasian population [112, 113].

Finally and more broadly, Calame et al recently highlighted the growing gap between the technical maturity of DECT and its limited clinical adoption [72, 114]. The authors emphasized that DECT becomes truly useful only when spectral biomarkers are standardized, consistently interpreted, and integrated into routine radiology reports. According to the authors, the major barrier is not technological but cultural, calling for a shift toward mandatory use of spectral information whenever spectral data are acquired.

## Future perspectives

### Artificial intelligence (AI)

AI has emerged as a promising revolution in medical imaging. In spectral CT, AI is primarily used for data analysis and reconstruction. Emerging AI-driven radiomics approaches are being explored for CRC imaging. Spectral CT has been increasingly integrated with radiomic analysis to enhance cancer detection, characterization, and treatment planning [115–118]. The authors concluded that deep learning algorithm reconstruction showed better subjective image quality, CNR and SNR of VMI_40_, without improving the detection rate of CRLM. Recently, in a population of 264 patients with biopsy-proven CRC, Feng et al investigated the value of radiomics analysis of DLCT-derived iodine maps for predicting preoperatively tumor deposits (TD+) (*n* = 80). The combined model incorporating the valuable clinical parameters and radiomics features demonstrated excellent performance in predicting TDs in CRC (AUCs of 0.926, 0.881, and 0.887 in the training, testing, and external validation cohorts, respectively), outperforming the clinical model in the training cohort and external validation cohorts and the radiomics model in two cohorts [119]. Wang et al used a radiomics model of the regional largest short-axis LN in 141 RC (58 LNM+ and 83 LNM-) to predict LNM, and found that the predictive radiomic model based on DECT had the highest diagnostic value, outperforming nIC and nZeff [25]. Lastly, Li et al developed a clinical-radiomics nomogram based on spectral CT to predict LNM in CRC, including 156 LN in 76 patients. The model, including age, CEA and radiomics signature of LN, reached an AUC of 0.879 and 0.824 in the training and validation set, respectively [120].

Regarding the reconstruction algorithm, Li et al explored deep learning image reconstruction at 40 keV in 35 patients with 164 CRLM and 55 benign liver lesions by evaluating CNR and SNR. The overall image quality, lesion conspicuity, and diagnostic confidence were subjectively evaluated to compare the differences in evaluation results among the different images [121]. Although no automatic segmentation method for CRC is currently available, AI is expected to enable automated segmentation of primary CRC in the near future, improving the applicability of predictive models based on spectral CT parameters.

### Spectral photon-counting CT

Spectral photon-counting CT (SPCCT) using energy-resolving detectors (i.e., photon-counting detectors) has emerged as a promising modality allowing improved spatial resolution, up to ~250 µm [16, 122, 123]. A potential for SPCCT in the imaging of CRC is the combination of spectral CT similar to DECT technology but with increased spatial resolution [124, 125]. Theoretically, this technique should help facilitate CT-based TNM staging thanks to its higher spatial resolution. Moreover, SPCCT improves tissue contrast with an energy beam similar to that used in EID-CT images, thanks to constant photon weighting, resulting in greater emphasis on the low-energy photons responsible for the photoelectric effect [126]. This could lead, in particular, to better detection of small CRC. Clinical applications remain limited, but potential uses in gastrointestinal imaging are beginning to emerge [125–127]. Surov et al evaluated in a pilot study of 41 patients the association between nIC, histopathology and tumoral response to NAC in RC. They found that nIC was higher in patients with LVI. Moreover, the authors found that nIC higher than 0.36 can predict good response (grade 2–4) to NAC [127].

## Conclusion

Spectral CT has demonstrated its potential as a non-invasive method in the prediction of CRC tumor aggressivity, staging, prognosis, histopathology characterization and mutation findings. Among spectral parameters, low-energy VMI, IC, nIC, and VMI slope appear to be the most relevant. When used alone or in combination with clinical data, tumor markers, and conventional CT, these parameters may enhance predictive models. However, the variability in acquisition techniques, parameter definitions, and measurement methods poses a challenge to result generalization. Among the various applications of spectral CT, some have already shown practical value in daily clinical workflows, particularly for detecting small liver metastases, evaluating peritoneal metastases, and improving staging of primary tumors. Other applications, mainly based on predictive models, remain promising but still largely experimental. These studies rely on small sample sizes and frequently lack a clear physiopathological rationale to explain the performance of their models, which limits their interpretability and clinical applicability. Integrating AI is expected to refine spectral measurement methodologies, enhance its performance and applicability of predictive models. Clinical impact on patient management should be further explored.

## Supplementary information

ELECTRONIC SUPPLEMENTARY MATERIAL

## Abbreviations

AEF: Arterial enhancement fraction
AP: Arterial phase
AUC: Area under the curve
CA19-9: Carbohydrate antigen
CEA: Carcinoembryonic antigen
CNR: Contrast-to-noise ratio
CRC: Colorectal cancer
CRLM: Colorectal liver metastases
CT: Computed tomography
DECT: Dual-energy computed tomography
DLCT: Dual-layer computed tomography
DP: Delayed phase
ECV*f*: Extracellular volume fraction
EDM: Early distant metastases
EMVI: Extramural vascular invasion
EP: Equilibrium phase
HER: Human epidermal growth factor receptor
HIPEC: Hyperthermic intraperitoneal chemotherapy
IC: Iodine concentration
IWR: Iodine water ratio
Ki-67: Antigen Kiel 67
KRAS: Kirsten rat sarcoma
LM: Liver metastasis
LN: Lymph node
LNM: Lymph node metastases
LVI: Lymphovascular invasion
MSI: Microsatellite instability
MSS: Microsatellite stability
MVD: Microvessel density
nIC: Normalized iodine concentration
nIWR: Normalized iodine water ratio
OS: Overall survival
PNI: Perineural invasion
RC: Rectal cancer
RECIST: Response evaluation criteria in solid tumors
ROI: Region of interest
SNR: Signal-to-noise ratio
SPCCT: Spectral photon-counting computed tomography
TD: Tumoral deposit
UN: Unenhanced CT
VEF: Venous enhancement fraction
VMI: Virtual monoenergetic images
VP: Venous phase
Zeff: Z-effective
