# Supplementary material for: Spectral CT imaging in colorectal cancer: current applications, limitations, and future perspectives
Source: Insights Imaging. 2026 Feb 9;17:39. doi: 10.1186/s13244-026-02212-9 (PMC12886653; doi:10.1186/s13244-026-02212-9)
Supplement: Supplementary file 1 — ELECTRONIC SUPPLEMENTARY MATERIAL [file 13244_2026_2212_MOESM1_ESM.pdf]

Spectral CT Imaging in Colorectal Cancer: current applications, limitations, and future perspectives

ELECTRONIC SUPPLEMENTARY MATERIAL

Supplementary 1 – An approx. 65-year-old male with right-sided colon cancer and suspicious synchronous CRLM on baseline evaluation.

DLCT (Iqon, Philips) in axial section at VP*.*

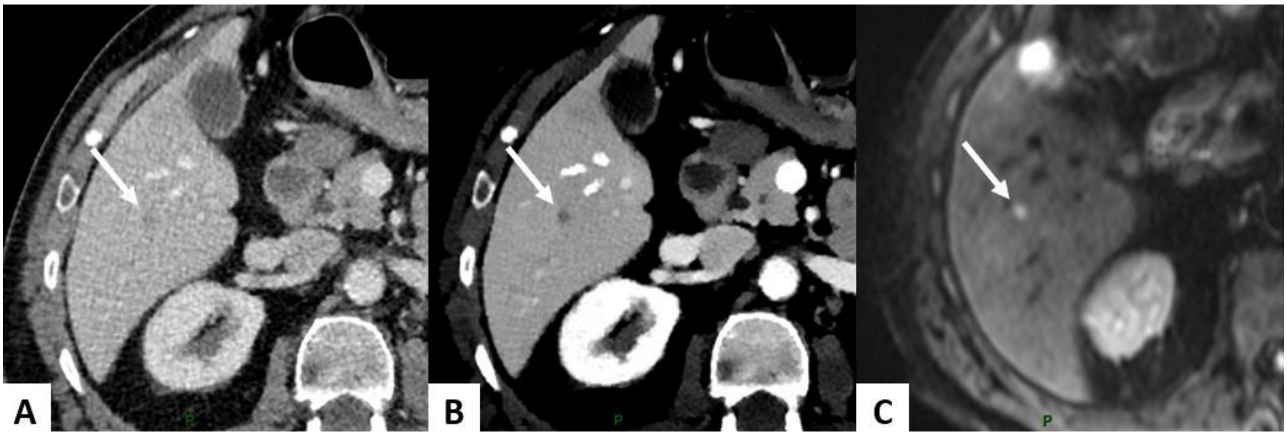

A. The conventional CT shows an equivocal hypodensity in segment VI of the liver. B. The 40 keV VMI clearly shows a well-defined lesion suspicious for CRLM. C. B-800 DWI liver MRI confirmed the suspicious restrictive hypersignal of the nodular lesion. Disappearance under chemotherapy confirmed LM.

Abbreviations: CRLM, colorectal liver metastases; CT, computed tomography; DLCT, dual-layer computed tomography; DWI, diffusion weighted imaging; LM, liver metastases; ROI, region of interest; VMI, virtual monoenergetic images; VP venous phase

Supplementary 2 – An approx. 70-year-old (A and B) and an approx. 55-year-old (C and D) male patient with metachronous PM from mucinous adenocarcinoma.

DLCT scan (Iqon, Philips) in axial section at VP.

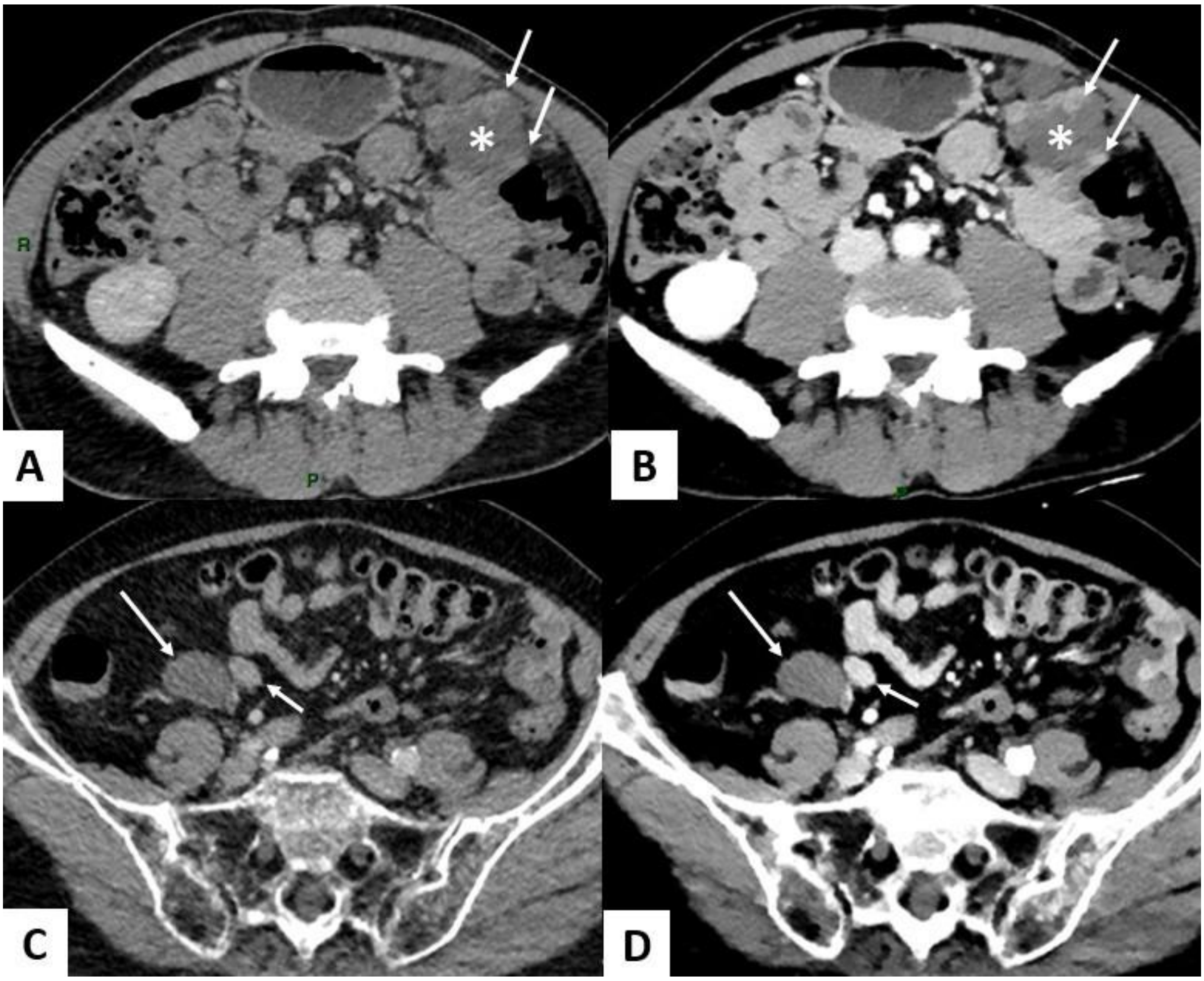

A. The conventional CT image shows a mucinous PM of the greater omentum, with low contrast between the solid components (arrows) and the central portion (asterisk). B. The 40 keV VMI image improves the contrast between the solid components (arrows) and the pseudo-liquid central portion (*). C. The conventional CT image also shows low contrast between a mucinous PM (long arrow) and the adjacent normal bowel (short arrow). D. The 40 keV VMI improves the contrast between the pseudo-liquid PM (long arrow) and the enhancing adjacent bowel loop (short arrow).

Abbreviations: CRS, cytoreductive surgery; CT, computed tomography; PM, peritoneal metastases; VMI, virtual monoenergetic images
